# Perceptions of US Adolescents and Adults With Sickle Cell Disease on Their Quality of Care

**DOI:** 10.1001/jamanetworkopen.2020.6016

**Published:** 2020-05-29

**Authors:** Julie Kanter, Robert Gibson, Raymona H. Lawrence, Matthew P. Smeltzer, Norma L. Pugh, Jeffrey Glassberg, Rita V. Masese, Allison A. King, Cecelia Calhoun, Jane S. Hankins, Marsha Treadwell

**Affiliations:** 1 Department of Medicine, University of Alabama, Birmingham, Birmingham; 2 Department of Hematology, Medical College of Georgia, Augusta University, Augusta; 3 Jiann Ping Hsu College of Public Health, Georgia Southern University, Statesboro; 4 School of Public Health, University of Memphis, Memphis, Tennessee; 5 RTI International, Research Triangle Park, North Carolina; 6 Mt Sinai School of Medicine, New York, New York; 7 Duke University School of Nursing, Durham, North Carolina; 8 Department of Pediatrics, Program in Occupational Therapy, Washington University School of Medicine, St Louis, Missouri; 9 Department of Medicine, Program in Occupational Therapy, Washington University School of Medicine, St Louis, Missouri; 10 Department of Surgery, Program in Occupational Therapy, Washington University School of Medicine, St Louis, Missouri; 11 Department of Pediatrics, Washington University School of Medicine, St Louis, Missouri; 12 Department of Hematology, St Jude Children’s Research Hospital, Memphis, Tennessee; 13 University of California, San Francisco, Benioff Children’s Hospital Oakland, Oakland

## Abstract

**Importance:**

Sickle cell disease (SCD) is the most common inherited red blood cell disorder in the United States, and previous studies have shown that individuals with SCD are affected by multiple health disparities, including stigmatization, inequities in funding, and worse health outcomes, which may preclude their ability to access quality health care. This needs assessment was performed as part of the Sickle Cell Disease Implementation Consortium (SCDIC) to assess barriers to care that may be faced by individuals with SCD.

**Objective:**

To assess the SCD-related medical care experience of adolescents and adults with SCD.

**Design, Setting, and Participants:**

This one-time survey study evaluated pain interference, quality of health care, and self-efficacy of 440 adults and adolescents (aged 15 to 50 years) with SCD of all genotypes and assessed how these variables were associated with their perceptions of outpatient and emergency department (ED) care. The surveys were administered once during office visits by trained study coordinators at 7 of 8 SCDIC sites in 2018.

**Results:**

The SCDIC sites did not report the number of individuals approached to participate in this study; thus, a response rate could not be calculated. In addition, respondents were not required to answer every question in the survey; thus, the response rate per question differed for each variable. Of 440 individuals with SCD, participants were primarily female (245 [55.7%]) and African American (428 [97.3%]) individuals, with a mean (SD) age of 27.8 (8.6) years. The majority of participants (306 of 435 [70.3%]) had hemoglobin SS or hemoglobin S β^0^-thalassemia. Most respondents (361 of 437 [82.6%]) reported access to nonacute (usual) SCD care, and the majority of respondents (382 of 413 [92.1%]) noted satisfaction with their usual care physician. Of 435 participants, 287 (66.0%) reported requiring an ED visit for acute pain in the previous year. Respondents were less pleased with their ED care than their usual care clinician, with approximately half (146 of 287 [50.9%]) being satisfied with or perceiving having adequate quality care in the ED. Participants also noted that when they experienced severe pain or clinician lack of empathy, this was associated with a negative quality of care. Age group was associated with ED satisfaction, with younger patients (<19 vs 19-30 and 31-50 years) reporting better ED experiences.

**Conclusions and Relevance:**

These results suggested that a negative perception of care may be a barrier for patients seeking care. These findings underscore the necessity of implementation studies to improve access to quality care for this population, especially in the acute care setting.

## Key Points

**Question:**

What are the barriers to care experienced by adolescents and adults living with sickle cell disease in the United States?

**Findings:**

This survey study conducted by the Sickle Cell Disease Implementation Consortium enrolled 440 adolescents and adults in 7 different states and found that most respondents were pleased with their usual care physicians but had negative experiences in acute care settings. Pain and the frequency of pain episodes were associated with patient-reported self-efficacy, further emphasizing the association of severe pain with poor outcomes in this population.

**Meaning:**

A negative perception of care in the emergency department setting may be a barrier for seeking care among adolescents and adults living with sickle cell disease.

## Teaser

This survey study evaluates pain interference, quality of health care, and disease-specific self-efficacy to assess perceived barriers for seeking care among adults and adolescents with sickle cell disease..

## Introduction

Improving access to health care and providing broad implementation of guideline-based health care services for individuals with sickle cell disease (SCD) are the ultimate goals of the Sickle Cell Disease Implementation Consortium (SCDIC), funded by the National Heart, Lung, and Blood Institute. The SCDIC aims to conduct multicenter studies using implementation science to accelerate the translation of science discoveries into clinical practice.^1^ One of the first consortium activities was to develop a patient-centered needs assessment to explore patient experiences with the quality and availability of health care and the associated patient-level factors. This information will be used to inform and guide the projects developed and implemented by the consortium. This article describes the results of this needs assessment and its relevance to future consortium activities.

Sickle cell disease is an inherited hemoglobinopathy and multisystem disease with acute complications, mainly painful vasoocclusive events, chronic progressive organ damage (eg, nephropathy and cardiomyopathy), and reduced life expectancy.^2^ In the United States, SCD affects approximately 100 000 individuals.^3^ Most are African American or Hispanic individuals and many are subject to disparities associated with geographic access to services and to low socioeconomic status.

Despite wide-ranging literature that explores barriers to care—including clinician- and system-driven barriers in SCD (eg, lack of qualified SCD clinicians,^4^ presence of disease, and racial/ethnic bias)^5,6^—patient-level experiences (eg, pain interference and self-efficacy) and patient perceptions of care quality have largely been overlooked. To our knowledge, the present survey study is the first comprehensive multiregional assessment of patient-level experiences.

Access to health care services is defined as the timely use of personal health services to achieve the best health outcomes.^7^ Three steps are critical in ensuring optimal access to health services in the United States: ingress into the health care system (eg, having adequate health care coverage), access to a location where needed health care services are provided, and identification of a clinician who the patient trusts and maintains communication with.^8^ The lack of available health care services (including knowledgeable clinicians) may lead to greater morbidity and even early mortality. Access to health care services is reported as suboptimal in SCD because many affected individuals are not receiving adequate treatment as outlined by evidence-based clinical guidelines.^9,10,11^ Adults with SCD often may not be seen by an SCD specialist, do not receive appropriate analgesia during vasoocclusive pain episodes, and are underprescribed disease-modifying therapies, such as hydroxyurea or long-term transfusion.^12,13^ Lack of access to health care services adds to the vulnerability of the SCD population, imparting greater risk of severe disease complications and early death.

Quality of health care can be measured or reported in a variety of ways. Indicators of quality in health care are traditionally used to assess measures of care delivery provided by clinicians. Historically (in SCD), these may include vaccination delivery^14^ or prescribing of antibiotics or disease-modifying agents.^15^ These indicators are often evaluated to identify target areas for improvement. From an individual’s perspective, patient satisfaction can also be used to measure the quality of care delivered. Although it is not clear that patient satisfaction equates to health outcomes, research suggests that patient evaluation of care is important to enhance patient engagement, to identify opportunities to improve health plans, and for benchmarking.^16^ Patient satisfaction is a complex paradigm that juxtaposes individuals’ health care experiences against their expectation of care.

Differences in treatment expectations between patients and clinicians may contribute to patient-level satisfaction.^17^ It is also important to note that it is often the interpersonal skills of the clinician—courtesy, empathy, and respect in addition to communication skills—that have been shown to be as essential as other technical skills, such as clinical competency and hospital equipment. The ultimate goal of the present study was to identify the individual-level impediments associated with obtaining health care services to inform the design of intervention strategies within implementation projects to be developed.

## Methods

### SCDIC Needs Assessment Process

The SCDIC is a cooperative research program comprising 8 academic or clinical sites and 1 data coordinating center distributed in locations across the US with high populations of individuals with SCD.^1^ The SCDIC activities are divided into 2 phases: a needs assessment (phase 1) and implementation studies (phase 2) with continuous registry enrollment. The needs assessment was initiated in the first year of the project to inform the design, development, and deployment of implementation studies. The needs assessment was designed as a concurrent mixed-methods^18^ approach including cross-sectional surveys, focus groups, and interviews with individuals with SCD and with clinicians. The present study assessed only the development and results of a survey of individuals with SCD. Our deployment of the needs assessment survey was aligned with best practices for survey research,^19^ including focusing on specific goals, generating a representative sample, matching questions to domains of interest, pilot testing the survey and procedures, training study coordinators in the conduct of the survey, and providing quality assurance. When designing the survey, we allowed respondents to skip questions that they were not comfortable answering (and continue the survey). Thus, response rates may differ by question (eTable in the Supplement). Institutional review board approval, including protection of participants’ confidentiality, was obtained by all sites. Once a person with SCD agreed to participate in this study, trained coordinators obtained informed consent from adults, and if the individual was a minor, from the legal guardian. Assent was obtained from adolescents between the ages of 15.0 and 17.9 years. Written informed consent or assent was obtained by research staff members of the respective SCDIC sites during routine clinic visits in accordance with the institutional guidelines based on the Declaration of Helsinki.^20^ Participants were compensated with gift cards for their involvement.

### Participant Selection

Individuals with SCD were eligible for this survey if they (1) had received a confirmed diagnosis of SCD (any genotype), (2) lived in the geographic region of 1 of the 8 sites that participated in this activity, (3) were between 15 and 50 years of age, and (4) were not experiencing acute symptoms of SCD. Unlike other SCDIC studies, this study permitted the inclusion of individuals up to 50 years of age to obtain more information regarding the needs of patients in this age group. The age limitations were specified by the request for application for this grant project. Although this was predominantly a convenience sample, efforts were made to achieve a broad reach from a heterogeneous group of the affected population. Participants were recruited through clinicians, websites, posted flyers, recruitment letters, health fairs, and clinical programs. Most participants were identified and recruited during clinical visits. Data collection occurred in the community or when participants were in an outpatient setting from July 2017 through March 2018. Table 1 gives characteristics of participating SCDIC sites, strategies for recruitment, and ultimate enrollment.

**Table 1.** Characteristics of Participating SCDIC Sites

Site	Estimated total No. of adults with SCD (≥18 y)	Estimated total No. of children with SCD (<18 y)	Inpatient services on site	Outpatient services on site	ED on site	Infusion center or ability to provide acute care in clinic	Primarily urban or rural practice	Survey site setting	Survey equipment	No. of participants enrolled
St Jude Children’s Research Hospital	400	850	Yes	Yes	Yes	Yes	Urban	Clinic	Tablet	101
Washington University	350	400	Yes	Yes	Yes	Yes	Urban	Community	Paper	19
Duke University	450	450	Yes	Yes	Yes	Yes	Urban	Clinic	Tablet	50
Augusta University	796	897	Yes	Yes	Yes	No	Rural and urban	Clinic	Tablet	163
Mount Sinai	734^a^	80^b^	Yes	Yes	Yes	Yes^c^	Urban	Clinic, inpatient	Tablet, paper	29
UCSF	708	276^d^	Yes	Yes	Yes	Yes	Urban	Clinic, community	Tablet, paper	55
MUSC	600	500	Yes	Yes	Yes	Yes	Rural and urban	Clinic	Tablet	86

Abbreviations: ED, emergency department; MUSC, Medical University of South Carolina; SCD, sickle cell disease; SCDIC, Sickle Cell Disease Implementation Consortium; UCSF, University of California, San Francisco.

^a^ Total number of unique adults older than 15 years seen across the Mount Sinai Health System, which includes 6 hospitals. Total at Mount Sinai Hospital = 381 (250 followed up in a clinic, 131 who were not followed up in a clinic but intermittently used acute care services).

^b^ Only children below 15 years of age.

^c^ Limited infusion capabilities for adults based on availability at the cancer center.

^d^ For a 5-county region, not just the clinic at UCSF. Within the region, patients had access to adult and pediatric inpatient, ED, and infusion units, but institutions varied throughout the region.

### Survey Domains Development

The needs assessment survey was designed to capture individual-level data using a combination of previously validated surveys collected using a standard approach across the SCDIC centers.^21^ Representatives from the 8 SCDIC clinical centers, the data coordinating center (RTI International), and the National Heart, Lung, and Blood Institute met regularly to develop measurement strategies for the needs assessment. Potential domains of interest were generated by the committee, and the final list of domains was determined through a consensus process.

The following criteria were applied to select the measures. Measures had to (1) be validated for use with patients with SCD, (2) address the domains of interest, and (3) allow for standardized administration in all participating sites. Once the final set of measures was assembled, patient consultants at 2 sites (North Carolina and California) beta tested the measures and provided the investigators with input about the relevance, understandability, and the amount of time required to complete the set of measures. The survey was then finalized and field tested with approximately 10 patients at each site without further changes and then broadly used in all participating sites (the full survey is available on request).

This needs assessment survey used patient-reported measures in 4 major domains: (1) pain interference and experience (health-related quality of life), (2) quality of health care, (3) social determinants of health, including numerous demographics, and (4) self-efficacy regarding SCD. These 4 domains were selected to evaluate specific areas of care, experiences, and coping that could be targeted by interventions in different geographic locations and for different age groups.

### Survey Procedures

Participants completed the survey in a private space immediately after providing informed consent or were scheduled for a separate visit at a convenient time and location. Participants with known or observed reading difficulties completed the survey as an interview. Participants could complete the survey on a tablet, with responses recorded directly in a Research Electronic Data Capture (REDCap)^22^ database. Alternatively, participants could complete a paper version, and results were then transferred to the REDCap database. Participants typically completed the survey in 15 to 20 minutes.

### Survey Measures

#### Demographic and Clinical Characteristics

Participants self-reported age, gender, race, ethnicity, and SCD phenotype using items from the consensus measures for Phenotypes and eXposures (PhenX) Toolkit, version 13.1, SCD Core demographic and clinical measures.^23,24^ Survey participants indicated the frequency of SCD pain episodes that they had experienced in the previous year, including the extent of any interference with usual daily activities but in the absence of health care use.

#### Pain Interference

Participants completed the Patient Reported Outcomes Measurement Information System (PROMIS) 4-item Pain Interference Short Form.^25^ PROMIS is an initiative funded by the National Institutes of Health to develop and validate patient-reported outcomes for clinical research and practice. Its measures have been developed and validated with state-of-the-science methods to be psychometrically sound and to transform how life domains are measured.^26^ The PROMIS Pain Interference measure has shown clinical relevance for adults with SCD.^7^

Participants in the present study indicated how much pain interfered with daily activities in the previous 7 days, including with work around the home, social activities, and household chores. Items were scored on a 5-point Likert scale, with 1 indicating “not at all” to 5 indicating “very much,” and total scores were generated by the HealthMeasures Scoring Service,^27^ a software application that automates and facilitates item response theory–calculated scoring of short-form data from HealthMeasures measurement systems, including PROMIS. Scores generated by the software included a T score with a mean (SD) of 50 (10). We used this T score for our analysis, with higher scores indicating more pain interference.

#### Quality of Care

We used a fixed-format questionnaire, the Adult Sickle Cell Quality of Life Measurement Information System (ASCQ-Me) quality of care (QoC) measure,^10^ to assess the health care experience of participants. Themes from the ASCQ-Me QoC formative research informed the construction of the ASCQ-Me QoC survey questions that were also modeled after the Consumer Assessments of Healthcare Providers and Systems (CAHPS) surveys.^28,29,30,31,32,33,34,35^

The ASCQ-Me QoC survey consisted of 27 questions, but skip patterns allowed respondents to complete the survey in as few as 5 questions if they did not have any SCD-related pain in the previous 12 months and had never sought emergency or ambulatory care. Thirteen ASCQ-Me QoC questions loaded on 3 domains of health care quality. The first domain, provider communication, consisted of 4 items mirroring the CAHPS provider communication domain; for example, “In the past 12 months, how often did the doctor or nurse listen carefully to you?” (rated never to always). The second domain, emergency department (ED) care, included 5 items regarding the patient’s interaction with staff (doctors, nurses, clerks, or receptionists) during emergency visits, including items that go beyond the CAHPS ED care domain by asking about the extent to which clinicians believed that the patient had severe pain and how successfully pain was treated in the ED. The third domain, access, consisted of 3 items, for example, “In the past 12 months, when you tried to make an appointment to see a clinician, how often were you able to get one as soon as you wanted?” (rated never to always). Although the CAHPS access to care only reflects access to outpatient care, the ASCQ-Me access composite included questions about access to both ED and outpatient care and a question about the influence of bad experiences on decisions to seek care.

Three questions addressed global evaluations of care by asking participants how often they were satisfied (never to always) with their usual clinician and with the QoC received from their usual clinician and from the ED. Participants also provided an overall evaluation of all the care they received on a scale from 0 to 10, anchored from worst to best care possible. Separate scores are produced for each of the 4 global questions. For the preceding 12 months, participants reported on the number of visits with their usual clinician, the number of ED visits for pain, and episodes of pain managed at home without going to a doctor, clinic, or hospital. Three other questions measure participants’ perceptions of how knowledgeable their primary care clinician was about SCD. The ASCQ-Me QoC composite scores have shown reliability (Cronbach α = 0.70-0.83), and QoC has been shown to be reliable, with good construct validity (*r*  =  0.32-0.83 correlations with global care ratings) and good precision for discriminating among groups experiencing poor QoC. In the ASCQ-Me QoC testing phase, adults with SCD reported worse care compared with adult Medicaid populations completing CAHPS.^10^

#### SCD Self-efficacy

The Sickle Cell Self-Efficacy Scale (SCSES)^36^ was used to assess self-efficacy for participants’ perceived ability to deal with daily aspects of SCD, such as pain and fatigue. The SCSES consisted of 9 items, with responses ranging from 1 (not at all sure) to 5 (very sure) on a Likert scale. Responses were summed so that higher scores indicated a higher degree of self-efficacy. The reliability and validity of the SCSES has been documented, with Cronbach α of 0.89 and significant positive correlations between SCSES scores and standardized measures of self-esteem, mastery, and locus of control.^36^ The SCSES has shown predictive validity, with higher self-efficacy correlated with fewer physician and ED visits.^36^ For adolescents with SCD, high levels of self-efficacy have been associated with fewer physical and psychological symptoms.^37^ In a systematic review of published papers between 2003 and 2013, studies that tested the association between self-efficacy and SCD outcomes showed positive correlations between self-efficacy during transition from pediatric to adult care and positive patient outcomes in the SCD population.^38^

### Data Collection and Storage

A REDCap database was housed at each SCDIC institution, with uniform data structure across sites for all elements.^22^ Deidentified participant-level data were shared from each site to the data coordinating center for centralized data analyses.

### Statistical Analysis

Summary statistics were calculated for all survey results, including frequency and percentage for categorical variables and mean, standard deviation, median, and interquartile range (IQR) for continuous variables. Age was evaluated as a continuous and as a categorical variable (<19, 19-30, and 31-50 years). Some individual measures had a smaller sample than the total number, in which case percentages are reported based on these smaller samples. We used univariate and multivariable models (linear regression for continuous outcomes and logistic regression for binary outcomes) to evaluate associations between demographic and disease characteristics and quality of care outcome variables. Multivariable models controlled for age group, gender, and SCD genotype as confounders and evaluated age group as an effect modifier with interaction terms. In addition to summary statistics, linear regression results included *P* values for overall associations. Logistic regression results included model-based odds ratio estimates with 95% CIs and *P* values for overall associations. The *P* values are reported at the .05 level with no adjustments for multiple comparisons.^39^ All analyses were conducted in SAS, version 9.4 (SAS Institute Inc).

## Results

### Participant Demographic and Clinical Characteristics

A total of 440 participants completed the survey and provided adequate responses for analysis. Sites did not report the number of individuals approached to participate in this study; thus, a response rate could not be calculated. Furthermore, respondents were not required to answer every question in the survey; thus, the response rate per question also differed for each variable. Sample sizes for each variable are reported in the eTable in the Supplement. Of 440 respondents, most were female (245 [55.7%]) and African American (428 [97.3%]) or non-Hispanic (418 [95.0%]) individuals, with a mean (SD) age of 27.8 (8.6) years (Table 2). A total of 128 of 371 respondents (34.5%) of the heads of household reported some college or an associate; degree. In addition, 131 of 433 respondents (30.3%) reported that they received disability income, but 50.8% were working (116 [26.8%]) or were students (104 [24.0%]). Few (84 of 433 [19.4%]) were married or living with a partner, and 268 of 404 respondents (66.3%) reported annual household incomes of less than $30 000. Of 424 respondents with health insurance, approximately one-third had more than 1 type of insurance. In total, 263 of 386 respondents (68.1%) reported receiving Medicaid (including some who had Medicaid in combination with Medicare or private insurance). Of 391 respondents, 137 (35.0%) reported receiving Medicare, and some of these participants also had other insurance types. The primary SCD diagnoses received by the respondents were hemoglobin (Hb) variant SS or Hb S β^0^-thalassemia (306 of 435; 70.3%).

**Table 2.** Patient Demographic and Clinical Characteristics

Characteristic	No. (%) of respondents
Total No.	440
Age, y	
≥18	92 (20.9)
19-30	176 (40.0)
31-50	172 (39.1)
Age, continuous, y	
Mean (SD)	27.8 (8.6)
Median (interquartile range)	28 (20-34)
Gender	
Male	193 (43.9)
Female	245 (55.7)
No response	2 (0.4)
Race	
African American	428 (97.3)
Other	8 (1.8)
No response	3 (0.7)
Ethnicity	
Hispanic, Latino, or Spanish	13 (3.0)
Not Hispanic, Latino, or Spanish	418 (95.0)
No response	9 (2.1)
Highest degree received, head of household	
≤12th Grade, no diploma	40 (10.8)
High school graduate or GED	93 (25.1)
Some college, AA degree	128 (34.5)
Bachelor’s degree or above	87 (23.4)
No response	23 (6.2)
Current employment status	
Working now	116 (26.8)
Other	80 (18.5)
Disabled	131 (30.3)
Student	104 (24.0)
No response	2 (0.5)
Marital status	
Married or living together	84 (19.4)
Other	340 (78.5)
No response	9 (2.1)
Annual income, $	
<9999	151 (37.4)
10 000-29 999	117 (29.0)
≥30 000	108 (26.7)
No response	28 (6.9)
Genotype	
Hb SS, Hb S β^0^-thalassemia	306 (70.3)
Hb SC disease	88 (20.2)
Hb S β^+^-thalassemia	18 (4.1)
Other variant	2 (0.5)
Don’t know	21 (4.8)
Health insurance	
Private health insurance	70 (16.5)
Medicare	129 (30.4)
Medicaid	166 (39.2)
Other	21 (5.0)
No coverage of any type	9 (2.1)
Don’t know	29 (6.8)

Abbreviations: AA, associate of arts; GED, General Educational Development; Hb S, hemoglobin variant S; Hb SC, hemoglobin variant SC.

### Pain Interference

Many participants experienced SCD-related pain. In the previous year, 287 of 437 participants (65.7%) reported requiring the ED for acute episodes of pain. In the previous 6 months, 168 of 437 participants (38.4%) reported 3 or more treat-and-release ED visits for pain, but 327 of 433 participants (75.6%) reported severe pain episodes at home for which they did not seek health care. In the previous 6 months, 210 of 327 participants (64.2%) reported at least 4 such pain episodes, and 207 of 323 participants (64.1%) also reported that they missed at least 1 week from usual activities because of pain at home. Although 147 of 436 participants (33.7%) reported no hospital admissions for pain in the previous 12 months, 151 of 436 participants (34.6%) reported 3 or more hospital admissions for pain during the same time frame.

The mean (SD) pain interference T score for participants in the present study was 59.2 (9.9) (median score, 61.3; IQR, 54.6-66.7; range, 41.6-75.6) suggesting a higher proportion of patients experiencing pain interference compared with the general population completing PROMIS pain measures.^39^ The mean (SD) SCSES score for participants in the present study was 30.8 (7.7) (median score, 31.0; IQR, 26.0-36.0; range, 9.0-45.0), similar to reference samples.^36^

Pain interference scores were associated with several important demographic factors (Table 3). Female participants (mean [SD]: females, 60.6 [9.6]; males, 57.3 [10.2]; *P* ≤ .001), participants who were married or living together (mean [SD]: married or living together, 61.8 [8.6]; others, 58.7 [10.1]; *P* = .01), and those receiving disability income (mean [SD]: disabled, 64.0 [7.3]; working, 57.6 [10.3]; student, 55.4 [11.1]; and others, 58.1 [9.0]; *P* < .001) all reported significantly higher levels of pain interference. Annual income was inversely associated with pain interference score (mean [SD]: earning <$9999, 60.9 [9.4]; earning $10 000-$29 999, 58.8 [10.3]; earning ≥$30 000, 57.8 [10.0]; *P* = .04). Participants with Medicare or Medicaid or with no insurance also reported higher pain interference scores. Reported numbers of pain episodes (mean [SD]: 0 episodes, 50.7 [9.8]; <4 episodes, 58.1 [9.4]; ≥4 episodes, 64.1 [6.9]), severe pain (mean [SD]: yes, 61.9 [8.4]; no, 50.7 [9.8]), ED use (mean [SD]: 0 visits, 53.7 [10.4]; 1-2 visits, 59.6 [9.2]; ≥3 visits, 63.8 [7.3]), hospitalization for pain (mean [SD]: 0 admissions, 53.9 [10.1]; 1-2 admissions, 59.6 [9.3]; ≥3 admissions, 63.9 [7.7]), and days of usual activity missed (mean [SD]: <1 week, 57.7 [8.6]; ≥1 week, 64.3 [7.3]) were all associated with pain interference scores (*P* < .001). When adjusted for age group, gender, and SCD genotype, findings associated with pain interference remained statistically significant for health care and pain-related covariates (Table 3).

**Table 3.** SCD Self-efficacy and Pain Interference

Covariate (No. missing from subgroup total)	SCD self-efficacy (n = 413)		Pain interference (n = 437)	
	Mean (SD)	*P* value	Mean (SD)	*P* value
Gender (2)				
Male	31.4 (8.1)	.21	57.3 (10.2)	<.001
Female	30.4 (7.3)		60.6 (9.6)	
Age, y (0)	30.8 (7.7)	<.001	59.2 (9.9)	<.001
Race (4)				
African American	30.9 (7.7)	.22	59.0 (9.9)	.02
Other	27.0 (6.0)		67.9 (4.3)	
Ethnicity (12)				
Hispanic	29.9 (7.5)	.70	63.5 (10.4)	.13
Not Hispanic	30.8 (7.7)		59.0 (9.9)	
Head of HH educational level (58)				
<HS	29.8 (9.2)	.12	59.3 (10.3)	.50
HS or GED or some college	30.2 (7.6)		59.7 (10.0)	
College graduate or professional	31.9 (7.5)		58.4 (10.1)	
Employment (9)				
Working now	32.9 (7.4)	<.001	57.6 (10.3)	<.001
Disabled	28.1 (7.1)		64.0 (7.3)	
Student	32.1 (7.6)		55.4 (11.1)	
Other	30.9 (7.4)		58.1 (9.0)	
Marital status (14)				
Married or living together	30.8 (7.4)	.89	61.8 (8.6)	.01
Other	30.9 (7.6)		58.7 (10.1)	
Annual income $ (55)				
<9999	28.8 (7.4)	<.001	60.9 (9.4)	.04
10 000-29 999	31.5 (7.6)		58.8 (10.3)	
≥30 000	32.5 (7.2)		57.8 (10.0)	
Medical insurance (38)				
Private	32.6 (6.8)	.06	57.2 (10.7)	.03
Medicare or Medicaid	30.3 (7.6)		60.2 (9.7)	
Other insurance	33.1 (7.4)		55.2 (10.1)	
No coverage	28.7 (8.1)		60.1 (11.6)	
Phenotype (23)				
SS or S β^0^	31.0 (7.7)	.42	59.3 (10.2)	. 83
SC	30.9 (7.5)		59.1 (9.7)	
S β^+^	30.6 (7.6)		57.5 (8.9)	
Other variant (n = 2)^a^	40.0 (0.0)		55.5 (4.1)	
No. of treated-and-released ED visits, 6 mo (0)^a^				
0	33.6 (7.8)	<.001	53.7 (10.4)	<.001
1-2	30.6 (6.9)		59.6 (9.2)	
≥3	28.4 (7.3)		63.8 (7.3)	
Severe pain, no health care, 6 mo (4)^a^				
Yes	29.8 (7.4)	<.001	61.9 (8.4)	<.001
No	33.8 (7.7)		50.7 (9.8)	
No. of pain episodes, 6 mo (104)^a^				
<4	32.7 (6.9)	<.001	58.1 (9.4)	<.001
≥4	28.2 (7.2)		64.1 (6.9)	
No. of pain episodes, 6 mo (4)^a^				
0	33.8 (7.7)	<.001	50.7 (9.8)	<.001
<4	32.7 (6.9)		58.1 (9.4)	
≥4	28.2 (7.2)		64.1 (6.9)	
No. of days missed usual activities, 6 mo (107)^a^				
<1 wk	32.9 (7.2)	<.001	57.7 (8.6)	<.001
≥1 wk	28.2 (7.0)		64.3 (7.3)	
No. of hospital admissions for pain, 12 mo (1)^a^				
0	33.5 (8.0)	<.001	53.9 (10.1)	<.001
1-2	31.1 (6.8)		59.6 (9.3)	
≥3	27.9 (7.0)		63.9 (7.7)	

Abbreviations: ED, emergency department; GED, General Educational Development; HH, household; HS, high school; SCD, sickle cell disease.

^a^ Covariate remained significant (for SCD self-efficacy and for pain interference) when adjusted for age group, gender, and phenotype.

### Quality of Care

The ASCQ-Me QoC was used to obtain data on usual (outpatient, nonacute) care and ED care. For the current sample, the ASCQ-Me QoC items loaded on 2 factors (provider communication and ED care) excluding an item asking about access to outpatient appointments (goodness-of-fit measures: root mean square error of approximation, 0.07; Bentler comparative fit index, 0.94). We reported composite scores (items that loaded onto the factors) and individual scores for both usual care and ED care.

### Usual Care (Defined as Outpatient Clinic Visit, Nonacute)

Of 437 respondents, 361 (82.6%) reported that they had a usual clinician for nonacute care. Of 361 respondents in this group, 300 (80.3%) reported their usual clinician typically treated “a lot of patients with SCD.” On the provider communication composite (Table 4),^40^ 76% reported that the provider explained things in a way they could understand, listened to them, and treated them with courtesy and respect. The 1 area in which the clinicians were not scored as highly was the ability to spend enough time with patients (66% rated their experiences as the most positive). The majority (314 of 341) of respondents (92.1%) noted they were “usually or always” satisfied with their usual clinicians, and 268 of 325 respondents (82.5%) were “usually or always” satisfied with care received during scheduled appointments. More than half (185 of 341; 54.3%) reported their usual clinician “very much” knew how SCD affected them personally.

**Table 4.** Quality of Care Composites

Composite	Composite score, %^a^		
	Never or sometimes	Usually	Always
Access to care composite^b^			
SCDIC needs assessment	41	28	31
ASCQ-Me field test	41	30	28
CAHPS adult Medicaid 2017	18	27	55
Clinician communication composite^c^			
SCDIC needs assessment	8	16	76
ASCQ-Me field test	12	23	65
CAHPS adult Medicaid 2017	9	17	74
Access to emergency department care^d^			
SCDIC needs assessment	55	26	19
ASCQ-Me field test	49	30	21
CAHPS adult Medicaid 2017	16	21	63

Abbreviations: ASCQ-Me, Adult Sickle Cell Quality of Life Measurement Information System; CAHPS, Consumer Assessments of Healthcare Providers and Systems; SCDIC, Sickle Cell Disease Implementation Consortium.

^a^ As stated in the *ASCQ-Me User’s Manual* “The interpretation of the composites is less straightforward than with the individual items, since the composite score is derived by combining responses from multiple individual items.”^40^^(p7)^ With that caveat, percentages for the composites (and 1 individual item) are summarized here. For consistency with the scoring guide, categories were labeled “never or sometimes, usually, and always,” where “always” defined respondents who reported the most positive response. Note, however, that not all items were scored on this scale.

^b^ For SCDIC and the ASCQ-Me field test populations, this composite was composed of the following items: “In the past 12 months, …when you tried to make an appointment to see DR/RN, how often were you able to get one as soon as you wanted; …when you needed care right away, how often did you get it as soon as you wanted; …what is the longest you had to wait before your pain was treated; and how important were bad experiences in the emergency room in your decision to avoid going for care.” For the CAHPS adult Medicaid 2017 population, this composite was composed of the following items: “How often was easy to get needed care, tests, or treatment”; and “got appointments with specialists as soon as needed.”

^c^ For SCDIC and the ASCQ-Me field test populations, this composite was composed of the following items: “In the past 12 months, …how often did this doctor or nurse explain things in a way that is easy to understand; …how often did this doctor or nurse listen carefully to you; …how often did this doctor or nurse treat you with courtesy and respect; and …how often did this doctor or nurse spend enough time with you.” For the CAHPS adult Medicaid 2017 population, this composite was composed of the following items: “Personal MD …explained things clearly; …listened carefully; …respected consumer comments; and …spent enough time with consumers.”

^d^ For SCDIC and the ASCQ-Me field test populations, this individual item was worded as “In the past 12 months, when you needed care right away, how often did you get it as soon as you wanted.” For the CAHPS adult Medicaid 2017 population, this item was worded as “Got urgent care for illness, injury, or condition as soon as needed.”

Questions about access to care were also posed to participants. Screening questions showed that 326 of 435 survey participants (74.9%) had attempted to make an appointment with a physician or nurse in the previous 12 months. In the composite analysis, when participants accessed outpatient health care, 76% were satisfied or somewhat satisfied with the time it took to get the physician or nurse appointment.

In logistic regression models, pain experience had a substantial negative association with satisfaction with scheduled nonacute appointments. Patients were less likely to be satisfied when they experienced severe pain (OR, 0.318 [95% CI, 0.093-0.823]; *P* = .03) or had 4 or more pain episodes (vs 0) in 6 months (OR, 0.237 [95% CI, 0.069-0.622]; *P* = .001) (Table 5).

**Table 5.** Patient Perceived Quality of Care

Covariate (No. missing of 413)	Satisfaction with regularly schedule appointment		Satisfaction with emergency department use	
	Odds ratio (95% CI)	*P* value	Odds ratio (95% CI)	*P* value
Gender (1)	0.932 (0.518-1.657)	.81	0.699 (0.435-1.121)	.14
Male	1 [Reference]		1 [Reference]	
Age, y (0)	1.006 (0.971-1.042)	.75	0.987 (0.959-1.015)	.35
1-Unit increase	1 [Reference]		1 [Reference]	
Race (4)	0.508 (0.106-3.608)	.43	1.286 (0.279-6.627)	.74
African American	1 [Reference]		1 [Reference]	
Ethnicity (11)	2.49 (0.513-9.766)	.21	2.729 (0.578-19.288)	.24
Hispanic	1 [Reference]		1 [Reference]	
Head of HH educational level (41)				
College graduate or professional	1 [Reference]	.52	1 [Reference]	.38
HS or GED	0.735 (0.363-1.460)		1.463 (0.856-2.513)	
<HS	0.584 (0.214-1.771)		1.288 (0.495-3.388)	
Employment (6)				
Working now	1 [Reference]	.92	1 [Reference]	.004
Disabled	0.919 (0.425-1.947)		0.34 (0.180-0.630	
Other	0.785 (0.328-1.911)		0.556 (0.259-1.182)	
Student	1.074 (0.447-2.661)		0.81 (0.396-1.656)	
Marital status (7)	1.032 (0.492-2.035)	.93	0.999 (0.552-1.806)	>.99
Married or living together	1 [Reference]		1 [Reference]	
Annual income, $ (37)				
≤9999	0.781 (0.349-1.692)	.36	0.784 (0.414-1.478)	.74
10 000-29 999	0.565 (0.250-1.236)		0.894 (0.455-1.749)	
≥30 000	1 [Reference]		1 [Reference]	
Medical insurance (23)				
Private	1 [Reference]	.36	1 [Reference]	.13
Medicare or Medicaid	0.575 (0.209-1.343)		0.564 (0.263-1.172)	
Other insurance	1.955 (0.300-38.487)		2.063 (0.516-10.483)	
No coverage	0.326 (0.055-2.618)		0.619 (0.101-3.784)	
Phenotype (19)				
SS or S β^0^	1 [Reference]	.44	1 [Reference]	.83
SC	0.61 (0.313-1.233)		0.827 (0.450-1.513)	
S β^+^	2.073 (0.388-38.384)		0.926 (0.251-3.420)	
Other	Undefined^a^			
No. of treat-and-release ED visits, 6 mo (0)				
0	1 [Reference]	.11	1 [Reference]	.12
1-2	0.633 (0.252-1.512)		0.431 (0.184-0.963)	
≥3	0.441 (0.189-0.939)		0.466 (0.206-1.001)	
Severe pain, no health care, 6 mo (2)^a^	0.318 (0.093-0.823)	.03	0.465 (0.222-0.931)	.03
No	1 [Reference]		1 [Reference]	
No. of pain episodes, 6 mo (57)^a^	0.306 (0.128-0.650)	.004	0.535 (0.304-0.934)	.03
<4	1 [Reference]		1 [Reference]	
No. of pain episodes, 6 mo (2)^b^				
None	1 [Reference]	.001	1 [Reference]	.01
<4	0.775 (0.198-2.593)		0.724 (0.314-1.623)	
≥4	0.237 (0.069-0.622)		0.388 (0.182-0.792)	
No. of days missed of usual activities, 6 mo (60)^c^	0.557 (0.266-1.097)	.10	0.394 (0.215-0.706)	.002
<1 wk	1 [Reference]		1 [Reference]	
No. of hospital admissions for pain, 12 mo (1)^c^				
0	1 [Reference]	.25	1 [Reference]	.01
1-2	0.517 (0.228-1.110)		0.385 (0.158-0.870)	
≥3	0.708 (0.315-1.508)		0.267 (0.111-0.593)	

Abbreviations: ED, emergency department; GED, General Educational Development; HH, household; HS, high school.

^a^ Covariate remained significant (for appointment satisfaction only) when adjusted for age group, gender, and phenotype.

^b^ Covariate remained significant (for both appointment satisfaction and emergency department satisfaction) when adjusted for age group, gender, and phenotype.

^c^ Covariate remained significant (for emergency department satisfaction) when adjusted for age group, gender, and phenotype.

### ED Care

Information about the location of the EDs used by participants was not collected. In total, 66% of respondents reported having had an ED visit in the previous 12 months. On the ED composite (Table 4), 29% reported having the most negative experience possible, 39% reported neutral experiences, and only 32% reported having the most positive experience possible. Overall, only half (146 of 287 [50.9%]) were “usually or always” satisfied with ED care received, and 52% indicated that bad experiences in the ED influenced their decisions to avoid seeking care. Only 26% of respondents were always satisfied with their ED care. Of 379 participants, 254 (67.0%) reported that they had delayed or avoided going to the ED when they thought they needed care in the previous 12 months. The ED composite analysis indicated that 47% of respondents gave the most negative ratings when asked if physicians seemed to “really care” about them; 35% gave the most negative ratings regarding whether nurses seemed to “really care” about them; 61% were neutral with regard to whether ED clinicians helped with their pain; and 50% were neutral when asked if clinicians believed how bad their pain was.

In logistic regression models, employment status was significantly associated with ED care satisfaction (Table 5). Compared with respondents who were currently working, participants who did not work and received disability income were 66% less likely to be satisfied with ED care. Students and those with “other” employment status (temporarily laid off, sick leave or maternity leave; looking for work, unemployed; retired; keeping house) also reported less satisfaction, although this finding was not statistically significant.

### ASCQ-Me QoC Compared With CAHPS

Table 4 gives the distribution of responses for the ASCQ-Me access to care composite compared with the 2017 CAHPS Clinician & Group Survey (N = 25 789) and the ASCQ-Me QoC field test sample (N = 561). Participants in the needs assessment rated their overall access to care as similar to participants in the ASCQ-Me field test. Both groups rated their overall access to care as the worst possible 41% of the time, whereas for the adult Medicaid sample, only 18% gave the worst possible rating. More than half of the adult Medicaid sample gave their overall access the best possible rating compared with approximately 30% of both the SCDIC and ASCQ-Me field test samples. Ratings of access to care to the ED contributed greatly to these ratings of overall access. The majority of both the SCDIC needs assessment and ASCQ-Me field test samples rated their access to ED care as the worst possible, whereas the majority of the adult Medicaid sample rated their access to ED care as the best possible. In addition, 45% were satisfied with the time it took to receive ED care, and 35% of patients experienced ED wait times of more than 2 hours.

### Sickle Cell Disease Self-efficacy

The associations with demographic and clinical characteristics on self-efficacy were evaluated with linear regression models (Table 3). There were no significant differences in SCD self-efficacy by gender, race, ethnicity, marital status, or disease phenotype. Older age, disabled employment status, and lower annual income were associated with significantly lower SCD self-efficacy scores. Significantly lower self-efficacy was also associated with more frequent pain episodes and frequency of severe pain episodes for which the individual did not seek treatment. Higher ED utilization rates and hospitalization rates for pain were also associated with lower SCD self-efficacy. Finally, participants with lower self-efficacy were more likely to miss usual activities because of disease complications. When adjusted for age group, gender, and SCD genotype, findings associated with self-efficacy remained statistically significant for health care and pain-related covariates.

### Age-Specific Results

We evaluated the association of each age group (<19, 19-30, 31-50 years) with the variables. Because the study included patients 15 to 18 years of age who were more likely to be treated in pediatric facilities and adults older than 18 years of age who were more likely to be treated in adult-focused facilities, it was necessary to evaluate these differences. There was no significant difference in the associations with satisfaction and usual appointments by age group. However, there was a notable difference in ED satisfaction by age group. Specifically, respondents who were younger than 19 years were more likely to be satisfied with ED care compared with those who were 31 to 50 years. We also found that the associations between ED satisfaction and degree of pain at home and between ED satisfaction and interference of pain with activities were modified by age group. Specifically, the association between severe pain at home without seeking health care and ED satisfaction differed significantly by age. Respondents who experienced severe pain without seeking health care and were younger than 19 years were more likely to be satisfied with ED care, whereas respondents who experienced severe pain without seeking health care and were aged 19 to 30 years were less likely to be satisfied with ED care. Respondents who managed severe pain at home and who missed doing their usual activities for at least 1 week were less likely to be satisfied with ED care. This finding did not differ across age groups.

### Site-Specific Results

There were no significant differences in appointment satisfaction, ED satisfaction, self-efficacy, or pain interference across the 7 sites participating in this study. Thus, results are consistent across sites and should be considered comprehensively.

## Discussion

This survey study represents the first multiregional needs assessment evaluation for SCD. The participants included individuals with SCD from different states, with different backgrounds, and across settings of care. However, when considering these results, it is important to acknowledge that these observations came from patients treated at some of the most prominent SCD treatment centers in the US. Although these patients’ individualized observations are made in the context of the relative availability of SCD specialty outpatient care, they can be used to highlight the importance of access to care. The results of these findings are being used in the development of the upcoming implementation trials for SCD. The novel findings of the present study include the significance of patient satisfaction with their usual clinicians despite concerns about their ED care.

It is also important to note the significant association of pain with self-efficacy. Many individuals within this participant group reported frequent pain episodes. Individuals with SCD were clearly concerned with their pain and the treatment of their pain. Our study is consistent with previous work that shows that many individuals experience high pain interference because of their SCD. Pain interference was also associated with the way individuals feel about the treatment they had received. However, although the frequency and severity of pain were associated with greater perceived care quality in the nonacute setting, this perception was not associated with self-efficacy or sociodemographic factors.

This is the first study, to our knowledge, to identify that a majority of adolescents and adults with SCD (82.6%) had access to nonacute, usual SCD care. However, these “usual care” clinicians were not always colocated in the same hospital where individuals received their acute care. In addition, ED physicians may not have had the opportunity to communicate with primary SCD physicians when individuals presented for acute painful events. A lack of communication and comanagement may have influenced respondents’ appraisal of ED care. We observed slightly fewer than half of respondents (49%) being satisfied with or perceived having adequate quality care within the ED setting. The experience in the ED of having severe pain or of feeling there was a lack of empathy for expressed pain was associated with a negative perceived quality of care. This negative perception of care in the ED is not unique to patients with SCD but shared with other patient populations with certain chronic conditions (ie, chronic obstructive pulmonary disease, congestive heart failure, asthma, and diabetes with diabetic ketoacidosis) who frequently visit the ED.^41,42,43,44,45,46^ Noticeably, patient satisfaction with ED care was higher in younger patients (<19 years) who were likely treated in pediatric facilities. Although unsurprising, it is certainly relevant to consider the differences in treatment of the same disease in different age-specific settings.

Overall, the ED findings are not new but are of interest when contrasted with the patient satisfaction in the nonacute environment. Furthermore, these results suggested the need to improve communication and comanagement to prevent these perceived negative experiences of care in the acute setting. These findings are therefore important when designing an implementation study to enhance care for affected individuals. A focus on both ensuring that individuals have access to an SCD specialist (usual clinician) and improving comanagement in the ED has therefore been a target of the implementation project.

Self-efficacy has not been well studied in previous SCD assessments. We found that self-efficacy was not associated with gender, race, ethnicity, or marital status. However, older individuals and those with disabled employment status (and lower annual income) had significantly lower SCD self-efficacy scores. This is especially important in recognizing the association of social determinants with health for this patient population. Self-efficacy was also notably associated with pain and with the frequency of pain episodes, further emphasizing the association of severe pain with poor outcomes in SCD. This finding is also important for showing why attention to pain management may facilitate both patient engagement and enhanced care engagement. Furthermore, this degree of pain interference and its association with self-efficacy is, to our knowledge, a novel finding. The finding is important for the development of strategies in which additional care management plans may be necessary for individuals who have lower self-efficacy to improve patient engagement.

Results from this needs assessment have been incorporated into the focus and design of the SCDIC implementation projects. One project is focused on individualizing the care experience in the ED to address concerns with consistent, meaningful, and rapid treatment of pain. Given the level of satisfaction with typical outpatient SCD care in this patient population, this study recognizes the importance of ensuring access to care for all affected individuals. Thus, another implementation project is exploring how best to identify and engage individuals who are not receiving adequate SCD care (unaffiliated patients). The third project is specifically examining strategies that can harness the positive patient-clinician relationship to increase hydroxyurea uptake and treatment adherence.

### Limitations

This study has several limitations. The primary limitation was the inability to generalize many of these findings to all individuals in this age group living with SCD in the United States. The majority of the participants in the present study had an identified clinician who was also an SCD specialist, which is not true for many individuals living with SCD. The survey components were chosen to specifically understand individual experiences with pain and with access to care to inform development of implementation strategies to overcome these barriers. In configuring the original survey, a minimum set of survey instruments was included as well as a menu of instruments that could be selected from to create an “enhanced” survey. Most sites selected the minimum survey to minimize participant burden, but then domains such as barriers to care and health literacy could not be assessed for the entire needs assessment sample. Furthermore, not every potential factor influencing outcomes could be assessed in one needs assessment (eg, health beliefs). Finally, this survey specifically assessed individual-level responses and did not include clinician- or system-level barriers to care. Those topics are addressed in separate forthcoming articles. In addition, more in-depth qualitative interviews were also performed with a subset of participants, which will also add additional valuable information to future articles.

## Conclusions

In conclusion, this study had several notable findings. It was clear that pain remained a barrier to self-efficacy for individuals with SCD in addition to affecting their quality of life. Many patients reported excellent communication and satisfaction with their usual clinician. However, care in the ED appeared to be lacking because of perceived lack of empathy from clinicians who provided acute care and may have limited disease-specific knowledge. Thus, many individuals with SCD may avoid seeking acute care because of noted concerns and poor responsiveness to their concerns, which may lead to poor outcomes. Attention is being paid to these issues in the upcoming SCDIC ED-focused implementation trial. Of paramount importance, however, is the level of satisfaction reported by patients who had an SCD physician. Unfortunately, many patients are without this benefit. As found in the present study, comfort with a usual clinician can enhance individuals’ perception of their quality of care and may provide the necessary relationship from which to improve outcomes. Thus, it is important that we use the SCDIC projects to identify and help individuals with SCD locate and engage with a knowledgeable clinician. It is equally important that strategies be implemented to enhance communication between the usual clinician and ED providers to improve trust, decrease stigma, and increase satisfaction with pain management.

## Notes

Supplement. **eTable.** Sample Sizes for Factors Associated With Study Outcomes (From Study Tables 3 and 5)
