# Supplementary material for: Perceptions of US Adolescents and Adults With Sickle Cell Disease on Their Quality of Care
Source: JAMA Netw Open. 2020 May 29;3(5):e206016. doi: 10.1001/jamanetworkopen.2020.6016 (PMC7260622; doi:10.1001/jamanetworkopen.2020.6016)
Supplement: Supplement. — eTable. Sample Sizes for Factors Associated With Study Outcomes (From Study Tables 3 and 5) [file jamanetwopen-3-e206016-s001.pdf]

# Supplementary Online Content

Kanter J, Gibson R, Lawrence RH, et al. Perceptions of US adolescents and adults with sickle cell disease on their quality of care. *JAMA Netw Open*. 2020;3(5):e206016. doi:10.1001/jamanetworkopen.2020.6016

**eTable.** Sample Sizes for Factors Associated With Study Outcomes (From Study Tables 3 and 5)

This supplementary material has been provided by the authors to give readers additional information about their work.

© 2020 Kanter J et al. *JAMA Network Open*.

**eTable.** Sample Sizes for Factors Associated With Study Outcomes (From Study Tables 3 and 5)

| Covariate | SCD Self-Efficacy (n=413)<br>n | Pain Interference (n=437)<br>n | Satisfaction with Regularly Scheduled Appointments (n=325)<br>n | Satisfaction with Emergency Department Utilization (n=287)<br>n |
|---|---|---|---|---|
| **Gender** | 411 | 435 | 324 | 286 |
| **Age, years** | 413 | 437 | 325 | 287 |
| **Race** | 409 | 433 | 321 | 284 |
| **Ethnicity** | 401 | 425 | 314 | 279 |
| **Head of HH Education** | 355 | 373 | 284 | 245 |
| **Employment** | 404 | 428 | 319 | 283 |
| **Marital Status** | 399 | 421 | 318 | 279 |
| **Annual Income** | 358 | 373 | 288 | 256 |
| **Medical Insurance** | 375 | 395 | 302 | 269 |
| **Phenotype** | 390 | 414 | 306 | 271 |
| **#Treat/Release ER visits, 6m** | 413 | 437 | 325 | 287 |
| **Severe pain, no healthcare, 6m** | 409 | 433 | 323 | 284 |
| **#Pain episodes, 6m** | 309 | 327 | 268 | 245 |
| **#Pain episodes, 6m (incl. those who responded 'No' to the lead-in question, i.e., 0 pain episodes)** | 409 | 433 | 323 | 284 |
| **#Days missed usual activities, 6m** | 306 | 323 | 265 | 241 |
| **Hospital admissions for pain, 12m** | 412 | 436 | 324 | 287 |

© 2020 Kanter J et al. *JAMA Network Open*.
